# Sequential model predictive direct speed control of PMSM

**DOI:** 10.1038/s41598-026-39256-2

**Published:** 2026-02-11

**Authors:** Lukáš Pancurák, Krisztián Horváth, Karol Kyslan

**Affiliations:** 1 Faculty of Electrical Engineering and Informatics, Dept. of Electrical Engineering and Mechatronics, Technical University of Košice, 042 00 Košice, Slovak Republic; 2 Faculty of Informatics and Electrical Engineering, Dept. of Electrical Engineering and Infocommunications, Széchenyi István University, 9026 Győr, Hungary

**Keywords:** Permanent magnet synchronous machine, Model predictive control, Sequential control, Finite control set, Electrical and electronic engineering, Mechanical engineering

## Abstract

Finite control set model predictive control (FCS-MPC) has emerged as a powerful strategy for permanent magnet synchronous motor (PMSM) drives. However, its performance strongly depends on appropriately chosen weighting factors, which directly affect control quality and, in some cases, may even lead to instability. Despite the crucial role of weighting factors, there is no systematic or generally accepted procedure for selecting their values, which limits the robustness and practical applicability of conventional FCS-MPC methods. To overcome this limitation, this paper presents the experimental validation of a sequential direct speed predictive control strategy for PMSM. The individual cost functions are evaluated sequentially, thereby tuning is simplified and weighting factors are reduced. Experimental results show that the original version of sequential direct speed control, as proposed in the literature, exhibits promising dynamic performance but suffers from instability and current ripples under certain conditions. To address these issues, an enhanced version of the sequential direct speed predictive control is proposed in the paper. It effectively suppresses instabilities and enhances the speed dynamic response of the drive. The proposed approach was experimentally validated using the OP 5600 rapid control prototyping platform running RT-LAB software and a 1.1 kW PMSM machine.

## Introduction

Permanent Magnet Synchronous Motors (PMSMs) are widely used in industrial applications due to their high efficiency, power and torque density, and dynamic performance. The most commonly used control method for PMSM is Field-Oriented Control (FOC), which relies on Proportional-Integral (PI) controllers in a cascade structure. However, PI control is fundamentally a linear approach applied to a nonlinear system, which can lead to performance limitations, particularly under transient conditions^1^. Direct Torque Control (DTC) has emerged as a solution to some of the limitations of FOC, while maintaining a simple control structure and improving dynamic performance^2,3^. However, this method still uses a PI controller in the outer loop for speed control. Certain limitations, such as restricted transient-state performance and torque fluctuations at low speed, persist due to the cascaded structure of this method^4^.

In recent years, Model Predictive Control (MPC) has gained attention as a promising alternative to traditional control methods, and has been widely applied in power electronics, drive systems, and power & energy applications^5–13^. In MPC, a system model is used to predict future states that are used to minimize cost function and select optimal control actions^5^. Different control objectives within the cost function are adjusted using weighting factors to fine-tune the performance of the controller^6^. Considering that MPC is an optimal and nonlinear control method^7^, it enables multi-objective control within a single control loop, eliminating the need for a cascaded structure and outer loop PI controllers. Simplicity, intuitive design, easy implementation, and seamless inclusion of constraints and nonlinearities without drawbacks, are among the many advantages of MPC. From the perspective of control objectives, one common approach to designing an MPC controller is to use it as a replacement for the inner-loop controller in traditional schemes. Examples of such implementations include predictive current, torque, or flux control schemes that retain a conventional outer-loop PI speed controller^8–10^, as well as more advanced variants, such as predictive control combined with nonlinear extended state observers, which mitigates steady-state current harmonics and improves control accuracy^14,15^. Advanced MPC scheme that incorporates the DC-link voltage as an explicit constraint in the cost function to suppress voltage oscillations in a film-capacitor-based PMSM drive was proposed in^16^. The inclusion of the voltage constraint provides effective system damping with reduced control set range. Although a faster dynamic response is achieved, the approach still retains the inherent drawbacks of a cascaded control structure and the use of a PI controller.

Multiple different methods were proposed to replace the outer-loop PI controllers with a different type of controllers, such as a deadbeat predictive controller with multi-timescale optimization as speed controller^11^ or sliding-mode controller^12^. As a further step toward simplifying the control architecture a Direct Speed Predictive Control (DSPC) emerged^1,17–19^ which completely eliminates the need for the outer-loop speed controller. This approach enables DSPC to achieve a cascade-free control structure, thereby simplifying the overall controller design. However, DSPC methods typically rely on weighting factors to balance multiple control objectives within the cost function, as a solution to multiobjective optimization problem (MOOP). The selection of these weighting factors is often empirical, time-consuming, and does not guarantee optimal control across different operating conditions. Some of the proposed solutions are either revolving around eliminating the weighting factors or adjusting them based on operating conditions^20^. This includes the use of two distinct cost functions, one for the transient state and another for the steady state^21^, or the implementation of more complex techniques, such as lexicographic optimization to solve the MOOP^22^. Another approach avoids weighting factors altogether by decoupling the control objectives, handled by a two-level hysteresis-based predictive controller^23^. Although these methods offer partial solutions to the issues outlined above, they often add complexity, increase computational burden, or may not be extended to direct speed control.

Another approach to address the issue of elimination of weighting factors is Sequential Predictive Control (S-PC), which evaluates control objectives in a stepwise manner, reducing the candidate switching states iteratively until the optimal voltage vector is selected^24–31^. Although the S-PC is a relatively recent method, it has already been widely adopted, although primarily for torque and flux control, while still using a PI controller for the outer-loop speed regulation. Despite this, it has already demonstrated promising results when compared to conventional methods that use traditional cost functions. This approach simplifies controller design while maintaining controller performance. However, as mentioned in multiple studies, challenges have emerged regarding the prioritization of the cost functions. In other words, determining which control objective should be addressed first is critical, as an incorrect choice can lead to controller instability^25,27^. Overall, this approach shows improved control performance, however, it still retains inherent drawbacks due to the cascade structure with a PI speed controller in the outer loop. Just very recently simulation study of Sequential Direct Speed Predictive Control (S-DSPC) has emerged^32^. This method eliminates weighting factors while taking advantage of the sliding manifold proposed in^18^, essentially helping speed error to converge faster, resulting in improved dynamic performance.

However, it should be noted that sequential evaluation inherently sacrifices global optimality, as decisions at earlier stages restrict the solution space for subsequent objectives, as analyzed in^33^. Nevertheless, such methods remain attractive for research, since they offer potential advantages, such as elimination of weighting factors, simplified implementation and a flexibility in design of additional control objectives^31^.

The motivation of this work is to overcome the practical challenges that currently limit the applicability of sequential direct speed predictive control in PMSM drives. Conventional DSPC requires tuning of multiple weighting factors, while sequential DSPC avoids weighting factors but has so far only been studied in simulation. This creates a need for experimental validation of sequential direct speed predictive control and to assess its performance. The main contributions of this paper can be summarized as follows:

- To the best of the authors’ knowledge, the first experimental validation of original sequential direct speed predictive control (S-DSPC): The method is validated by laboratory experiments with 1.1 kW PMSM drive, thus extending previous simulation-only study^32^.
- Comparison with conventional DSPC: Experimental results show that S-DSPC achieves promising dynamics with simpler tuning, but may suffer from instability and current distortions.
- Proposal of an enhanced S-DSPC (ES-DSPC): By modifying the cost function with a speed-scaling term and refining the sequence of objective evaluation, the enhanced method eliminates instabilities, supports larger sliding manifold constants, and improves the speed dynamic response. The THD values obtained are, however, relatively high. This is an inherent limitation of the presented method, as it was not primarily explicitly optimized for harmonic reduction.

The paper is structured as follows. First, the mathematical model of the permanent magnet synchronous machine (PMSM) is presented, together with the discrete-time predictive model used for state prediction and the sliding-mode load-torque observer employed for disturbance estimation. Next, the conventional direct speed predictive control (DSPC) strategy is reviewed to establish a reference framework. This is followed by a comprehensive description of the proposed sequential DSPC scheme and its enhanced variant, including the formulation of the cost functions, the sequential objective evaluation procedure, and the overall control algorithm. Subsequently, simulation results comparing the conventional DSPC and the enhanced sequential DSPC methods are presented and analyzed using multiple quantitative performance metrics. The experimental setup is then described in detail, and the experimental results are provided to validate the proposed control approach under practical operating conditions. Finally, the paper concludes with a summary of the main findings and discusses directions for future research.

## Mathematical models of PMSM and inverter

### Model of surface-mounted PMSM

The continuous time model of surface-mounted PMSM (SM-PMSM) in synchronous rotating reference frame can be expressed as follows:

1 $$\begin{aligned} \frac{di_d}{dt}&= \frac{1}{L_s}(u_d - R_s i_d + \psi _q\omega _e), \end{aligned}$$

2 $$\begin{aligned} \frac{di_q}{dt}&= \frac{1}{L_s}(u_q - R_s i_q - \psi _d\omega _e), \end{aligned}$$

3 $$\begin{aligned} \frac{d\omega }{dt}&= \frac{1}{J}(T_e - T_L - B\omega ), \end{aligned}$$

where:

$$\begin{aligned} \psi _d&= L_si_d + \psi _{pm}, \\ \psi _q&= L_si_q. \end{aligned}$$

Since for surface-mounted PMSM the reluctance remains the same along both the $d$-axis and $q$-axis, resulting in identical inductances ($L_q = L_d = L_s$), the torque can be expressed as:

4 $$\begin{aligned} T_e = \frac{3}{2}p\psi _{pm}i_q, \end{aligned}$$

where $u_d$ and $u_q$ are the *d*- and *q*-axis voltages [*V*], $i_d$ and $i_q$ are the *d*- and *q*-axis currents [*A*], $\omega _e$ is the electrical angular velocity [*rad*/*s*] ($\omega _e = p\omega$), $\omega$ is the mechanical angular velocity [*rad*/*s*], *p* is the rotor pole pairs, $\psi _d$ and $\psi _q$ are the *d*- and *q*-axis components of the stator flux vector [*Wb*], $\psi _{pm}$ is the permanent magnet flux linkage [*Wb*], $R_s$ is the stator resistance [$\Omega$], $L_s$ is the stator inductance [*H*], $T_e$ is the electrical torque [*Nm*]; $T_L$ is the load torque [*Nm*], *B* is a linear viscous friction coefficient [*Nms*/*rad*] and *J* is the total inertia recalculated on the motor side [$kg m^2$].

### Inverter model

A two-level voltage source inverter (2L-VSI) is used to generate stator voltage from DC line voltage. The switches in each phase operate in complementary manner, with states represented by $S_a$, $S_b$, $S_c$, determining the output voltage:

5 $$\begin{aligned} v_s = \frac{2}{3}V_{dc}(S_a + S_be^{\frac{j2\pi }{3}} + S_ce^{\frac{j4\pi }{3}}), \end{aligned}$$

where $V_{dc}$ is DC link voltage. The 2L-VSI generates eight discrete voltage vectors: six active vectors ($u_1 - u_6$) and two zero vectors ($u_0$ and $u_7$). The vector magnitudes and their corresponding switching states are presented in Table 1.

### Predictive model of PMSM

To implement the model-predictive controller, future values of the machine’s current, speed, and torque must be predicted. These predictions are obtained from the discrete-time model of the PMSM. Mechanical equations (3) and (4) are discretized using a second-order Taylor series expansion, while the current equations (1) and (2) are discretized using a first-order Taylor series expansion. Second-order discretization is necessary for the mechanical equations to ensure that the $d,q$ voltages explicitly appear in the resulting discrete equations. This yields the following discrete model of PMSM:

6 $$\begin{aligned} i_d^p(k+1)&= a_1i_d(k)+a_2\omega (k)i_q(k) + a_3u_{di}(k), \end{aligned}$$

7 $$\begin{aligned} i_q^p(k+1)&= a_1i_q(k)-a_2\omega (k)i_d(k) - a_4\omega (k) + a_3u_{qi}(k), \end{aligned}$$

8 $$\begin{aligned} \omega ^p(k+1)&= a_5 \omega (k) + a_6 i_q (k) + a_7 \hat{T}_L(k) + a_8 \omega (k) i_d(k) + a_9 u_{qi}(k) , \end{aligned}$$

9 $$\begin{aligned} T_e^p(k+1)&= \frac{3}{2}p\psi _{pm} i_q^p(k+1), \end{aligned}$$

**Table 1:** Possible Switching States and Generated Voltage Vectors of Two-Level Voltage-Source Inverter.

Switching State				Voltage Vector	
	$S_a$	$S_b$	$S_c$	$u_\alpha$	$u_\beta$
$u_0$	0	0	0	0	0
$u_1$	1	0	0	$2V_{dc}/3$	0
$u_2$	1	1	0	$V_{dc}/3$	$\sqrt{3}V_{dc}/3$
$u_3$	0	1	0	$-V_{dc}/3$	$\sqrt{3}V_{dc}/3$
$u_4$	0	1	1	$-2V_{dc}/3$	0
$u_5$	0	0	1	$-V_{dc}/3$	$-\sqrt{3}V_{dc}/3$
$u_6$	1	0	1	$V_{dc}/3$	$-\sqrt{3}V_{dc}/3$
$u_7$	1	1	1	0	0

where $u_{di}$, $u_{qi}$ represent the voltages corresponding to all possible switching states, as listed in Tab. 1, and $\hat{T}_L$ is the estimated value of the load torque, which is discussed in more detail in the following subsection. The constants $a_1$-$a_9$ represent model parameters and are calculated as follows:

10 $$\begin{aligned} a_1&= 1 - \frac{T_s R_s}{L_s}, \end{aligned}$$

11 $$\begin{aligned} a_2&= T_s p, \end{aligned}$$

12 $$\begin{aligned} a_3&= \frac{T_s}{L_s}, \end{aligned}$$

13 $$\begin{aligned} a_4&= \frac{T_s \psi _{pm} p }{L_s}, \end{aligned}$$

14 $$\begin{aligned} a_5&= 1 - \frac{T_s B}{J} - \frac{a_{10}\psi _{pm} p T_s^2}{2J} + \frac{a_{11} B T_s^2}{2}, \end{aligned}$$

15 $$\begin{aligned} a_6&= \frac{3T_s p \psi _{pm}}{2J} - \frac{a_{10}R_s T_s^2}{2J} - \frac{a_{11}3p \psi _{pm} T_s^2 }{4}, \end{aligned}$$

16 $$\begin{aligned} a_7&= - \frac{T_s}{J} + \frac{a_{11}T_s^2}{2}, \end{aligned}$$

17 $$\begin{aligned} a_8&= - \frac{a_{10}pL_s T_s^2}{2J}, \end{aligned}$$

18 $$\begin{aligned} a_9&= \frac{a_{10}T_s^2}{2J}, \end{aligned}$$

where

19 $$\begin{aligned} a_{10}&= \frac{3p\psi _{pm}}{2L_s}, \end{aligned}$$

20 $$\begin{aligned} a_{11}&= \frac{B}{J^2}, \end{aligned}$$

and $T_s$ is the sampling time. The coefficients (10)-(18) are utilized in the discrete model equations (6)-(8) for predicting the machine states. The robustness of this discrete predictive model against parameter variations was verified in^34^, where the same predictor equations were applied within a sequential direct speed predictive control scheme. The study confirmed that the controller remained stable under variations in stator resistance, inductance, inertia, and flux, thereby validating the applicability of the model.

### Sliding-mode load torque observer

As evident from (8), the accurate operation of the predictive model relies on knowing the value of the load torque. Since this information is typically not available or measurable, the load torque must be observed. Several methods can be applicable for load torque observation, including the Luenberger observer^35^, Kalman filter^4,19^, or sliding-mode observers^36,37^. The primary goal in selecting a specific type of load torque observer was to preserve the simplicity of the control structure while ensuring that control performance was not adversely affected. Based on the literature^36,37^, where the sliding-mode observer was paired with the predictive controller, it was determined that the sliding-mode observer offers the best compromise due to its relatively simple design and low computational requirements combined with robust behavior. Therefore, the sliding mode load torque observer (SMLTO) was implemented to estimate the load torque in this paper, based on mechanical variables of PMSM. The SMLTO is derived from the torque equation (4) and motion equation of PMSM, resulting in the state space equation describing the mechanical part of PMSM:

21 $$\begin{aligned} \begin{bmatrix} \dot{\omega } \\ \dot{T}_L \end{bmatrix} = \begin{bmatrix} -\frac{B}{J} & -\frac{1}{J} \\ 0 & 0 \end{bmatrix} \begin{bmatrix} \omega \\ T_L \end{bmatrix} + \begin{bmatrix} \frac{3p\psi _{pm}}{2J} \\ 0 \end{bmatrix} i_q. \end{aligned}$$

Based on state-space model given by (21), after substituting the motor variables into the general sliding mode observer equation, the discrete SMLTO takes the following form:

22 $$\begin{aligned} \begin{bmatrix} \hat{\omega }(k+1) \\ \hat{T}_L(k+1) \end{bmatrix} = \begin{bmatrix} 1 -\frac{B T_s}{J} & -\frac{T_s}{J} \\ 0 & 1 \end{bmatrix} \begin{bmatrix} \hat{\omega }(k) \\ \hat{T}_L(k) \end{bmatrix} + \begin{bmatrix} \frac{3p\Psi _{PM} T_s}{2J} \\ 0 \end{bmatrix} i_q(k) + \begin{bmatrix} 1 \\ m \end{bmatrix} \xi \Big (\omega (k) - \hat{\omega }(k) \Big ), \end{aligned}$$

where $\hat{\omega }$ is estimated mechanical speed, $\hat{T}_L$ is estimated load torque, *m* is a feedback gain of the SMLTO and $\xi$ is the sliding mode control function of the speed estimation error. The feedback gain must be negative ($m < 0$) to ensure that estimation error will converge to zero^38^. The estimated load torque $\hat{T}_L$ is then fed into the control algorithm of sequential model predictive control.

## Conventional direct speed predictive control

This section describes the conventional direct speed predictive control of PMSM shown here as it will serve as a reference for comparison with our proposed sequential MPC method. The finite control set MPC (FCS-MPC) controller was designed, similar to the one previously published in^19^. However, in practical implementation of the predictive control, finite computation time must also be taken into account. It introduces a delay between state measurement and the application of the selected switching vector, due to the time required to compute predictions and evaluate the optimization algorithm. If this delay is not considered, the control action is based on outdated system information, which degrades dynamic response and increases current ripple. To achieve better controller performance, computational delay compensation, using two-step prediction compensation (TSC), was implemented, in the way described in^19,39^. The TSC approach compensates for this effect by predicting the system state two sampling steps ahead: the first step accounts for the computational time, while the application of the switching states is moved to the beginning of the next sampling interval. This aligns the prediction with the actual actuation instant, leading to improved prediction accuracy.

Optimization criterion for this control method can be defined by following cost function:

23 $$\begin{aligned} J&= \underbrace{\lambda _\omega \big (\omega ^*(k)-\omega ^p(k+2)\big )^2}_a + \underbrace{\lambda _{i_d} \big (i_d^P(k+2)\big )^2}_b + \underbrace{\lambda _{i_q} \big (i_q^*(k+2) - i_q^p(k+2)\big )^2}_c + \underbrace{f\big (i_d^p(k+2),i_q^p(k+2)\big )}_d, \end{aligned}$$

where reference values are denoted by superscript “*”, and predicted values by “p” superscript. The control objectives described by each term of the cost functions can be summarized as follows:

- ensures tracking of the speed reference $\omega ^*$,
- ensures a maximum torque-per-ampere optimization,
- ensures a tracking of torque generating current $i_q^*$,
- provides a limitation maximal current,

where terms *a*, *b* and *c* are weighted by weighting factors $\lambda _\omega$, $\lambda _{i_d}$ and $\lambda _{i_q}$, respectively. The nonlinear function, described by term *d*, for limiting the amplitude of the stator current vectors, is defined as:

24 $$\begin{aligned}&f \left( i_d^p(k+2), i_q^p(k+2) \right) = {\left\{ \begin{array}{ll} \infty , & \text {if } \left| i_s^p(k+2) \right|> i_{\max }, \\ 0, & \text {if } \left| i_s^p(k+2) \right| \le i_{\max }, \\ \end{array}\right. } \end{aligned}$$

where $i_s^p$ is vector of current magnitude and is calculated as $i_s = \sqrt{{i_d^p}^2 + {i_q^p}^2}$. For clarity, the overcurrent protection (24) (term *d*) will be denoted as $i_{oc}^p$ in the remainder of the paper. The resulting DSPC control algorithm consists of the following steps:

1. measurement of phase currents and angular position,
2. application of selected switching states to the inverter.
3. calculation of controlled variables ($i_d^p, i_q^p, \omega ^p$) for the next sampling instant $t_{k+1}$, based on just applied voltage vectors $u_d(k)$,$u_{q}(k)$
4. estimation of mechanical load torque $\hat{T}_{L}$ using SMO,
5. prediction of controlled variables ($i_d^p, i_q^p, \omega ^p$), using estimated values, for the $t_{k+2}$ sampling instant, for all possible inverter switching states, given by voltage vectors $u_{di}$ and $u_{qi}$,
6. evaluation of the cost function *J* for each prediction,
7. selection of optimal actuation,

Detailed description of conventional DSPC can be found in^18,19^.

## Sequential direct speed predictive control

### Sequential DSPC

Compared to the conventional DSPC described in the previous section, which relies on a single cost function consisting of multiple control objectives, S-DSPC evaluates a series of cost functions, each dedicated to a specific control objective. These cost functions are processed sequentially. In each step, the worst-performing voltage vector candidates are eliminated based on the predicted and reference values relative to the given control objective. This progressive elimination of suboptimal candidate vectors, and consequently the reduced number of required predictions, provides an opportunity to simplify the algorithm. As each sequence considers only a single control objective, there is no need for the weighting factors to be implemented, eliminating the need for weight tuning. This is especially advantageous given the absence of a universally applicable method for weight tuning.

The following cost functions, as proposed in^32^, were formulated for experimental verification with TSC implemented:

25 $$\begin{aligned} J_{\omega }&= \big (\omega ^*(k) - \omega ^p(k+2)\big )^2 + i_{oc}^p(k+2), \end{aligned}$$

26 $$\begin{aligned} J_{i_d}&= \big (i_d^p(k+2)\big )^2 + i_{oc}^p(k+2), \end{aligned}$$

27 $$\begin{aligned} J_{T_e}&= \mid S \mid + i_{oc}^p(k+2). \end{aligned}$$

**Fig. 1:**
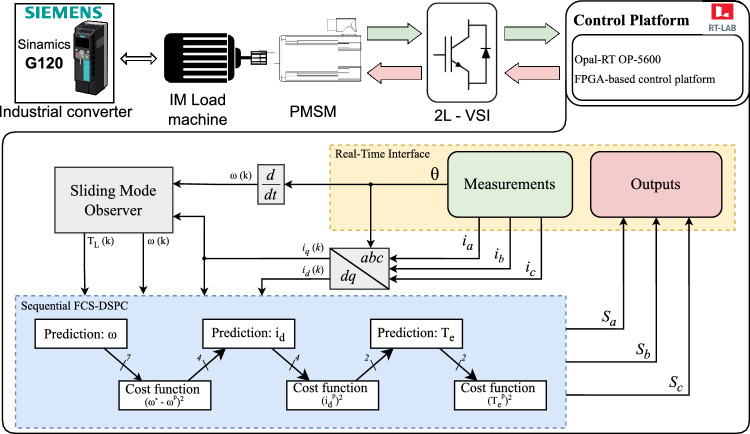
Control diagram of proposed sequential DSPC.

The proposed control scheme is shown in Fig. 1. The cost functions are evaluated iteratively. At each stage, the candidate set is reduced to about half its size, starting from seven vectors and narrowing down to the final voltage vector. Given that inertia, and therefore the mechanical time constant is significantly higher than the electrical time constant, it was determined that the most effective approach is to first evaluate the cost function $J_{\omega }$ and select the four vectors with the lowest error values. The selected vectors are then evaluated in the next sequence using the cost function $J_{i_d}$, for maximum torque per ampere ratio. The two vectors with the lowest error values are then selected to proceed to the following sequence. Note, that it is necessary to calculate $i_d$ current predictions only for four selected vectors, as three vectors were already eliminated in the previous sequence. In the final sequence, the optimal voltage vector is selected based on the cost function (27), where optimal torque control is the primary selection criterion. The choice of the elimination sequence (7$\rightarrow$4$\rightarrow$2$\rightarrow$1) was not arbitrary. Based on the different time scales of the mechanical and electrical dynamics, the $J_\omega$ cost function was prioritized in the elimination process. Also, through systematic testing of various alternative patterns, we found that only this configuration consistently ensured reliable operation, whereas other combinations resulted in slower dynamics, infeasible real-time implementation, or even a failure of the algorithm.

Instead of the conventional torque control term, a sliding manifold, as proposed in^18^, was implemented to improve speed control performance. The sliding manifold term $S$ is defined as a function of speed and torque error as follows:

28 $$\begin{aligned} S = ce_\omega - e_{T_e}, \end{aligned}$$

where

29 $$\begin{aligned} e_\omega&= \omega ^*(k) - \omega ^p(k+2), \end{aligned}$$

30 $$\begin{aligned} e_{T_e}&= T_e^p(k+2) - \hat{T}_L(k). \end{aligned}$$

The MPC can naturally drive error terms in the cost function to approach zero. However, the convergence rate of the sliding manifold term is much faster than that of the speed error term. As a result, the sliding manifold term $S$, described in (28) converges to zero faster when the speed changes. This is because $S$ is a linear combination of the speed error (29) and torque error (30), as proposed in^18^. The parameter $c$ shapes the structure of the sliding manifold rather than balancing multiple objectives. In this formulation, the sliding-manifold term addresses a single objective related to torque dynamics, while $c$ merely adjusts the dynamic coupling and adds an additional degree of freedom for adjusting the transient response. This parameter should be selected according to the difference between the mechanical and electrical time constant. The resulting value should be greater than zero. A higher value leads to a faster dynamic response but also increases fluctuations in the measured values.

### Enhanced sequential DSPC

The experimental verification of S-DSPC, described in the previous subsection, revealed issues with controller instability and significant current ripples, particularly for increased sliding manifold constant *c*. To mitigate these effects, the coefficient *c* for S-DSPC had to be reduced significantly, which constrained achievable dynamic performance of the controller, by limiting the speed of error convergence and slowing the transient response.

Upon investigating potential solutions, we found that controlling $d$-axis current $i_d$ in the first term improves the overall performance. As a result, the cost function was modified as follows:

31 $$\begin{aligned} \begin{aligned} J_{\omega }&= \big (\omega ^*(k) - \omega ^p(k+2)\big )^2 + \frac{|\omega ^*|}{\omega _n}\big (i_d^p(k+2)\big )^2 + i_{oc}^p(k+2), \\ J_{i_d}&= \big (i_d^p(k+2)\big )^2 + i_{oc}^p(k+2), \\ J_{T_e}&= \mid S \mid + i_{oc}^p(k+2). \end{aligned} \end{aligned}$$

In the first cost function, the term $\tfrac{|\omega ^*|}{\omega _n}$ represents the speed scaling factor, where $\omega _n$ is the nominal speed of the motor in [rad/s]. Speed scaling factor adjusts the weighting of the $i_d$ current to ensure that the control objective dynamically adapts to changes in the reference speed. To provide a clear understanding of the finalized implementation of the S-DSPC control scheme, the sequential cost function evaluation and candidate vector selection procedure is summarized in the Algorithm (1).

**Algorithm 1:**
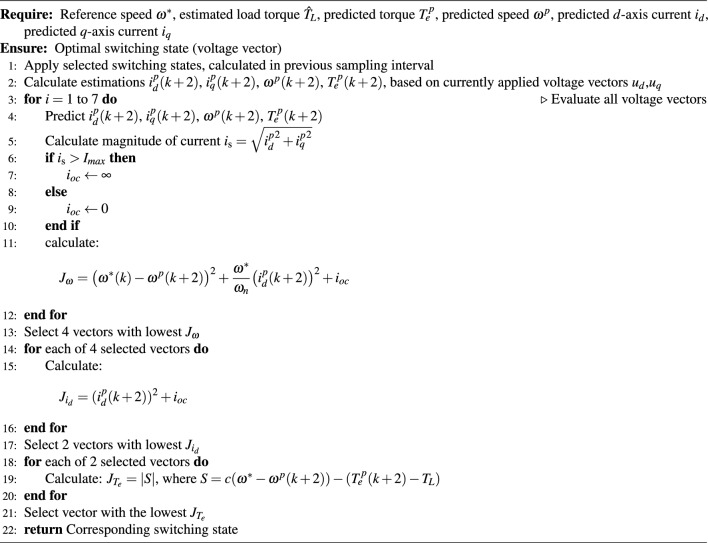
Pseudo-algorithm of Enhanced Sequential DSPC (ES-DSPC).

## Simulation results

**Fig. 2:**
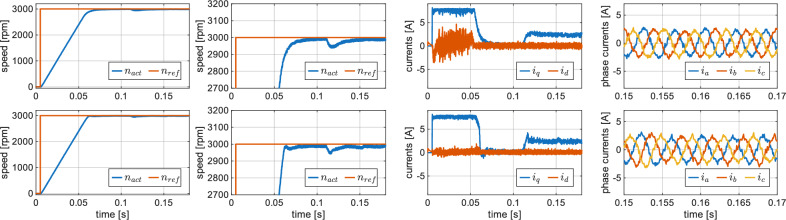
Simulation results, comparison of C-DSPC (top row figures) and ES-DSPC (bottom row figures) for rated load.

Both ES-DSPC and C-DSPC methods were simulated in MATLAB/Simulink using a Simscape-based PMSM model, with the inverter transistors represented as ideal semiconductor switches. The sampling time was set to $25~\mu$s and the fundamental sample time to $1~\mu$s. The simulation results for a step change of the speed reference to the nominal value, followed by a step application of the nominal load torque, are presented in Fig. 2. To enable a fair comparison, both methods were tuned to achieve identical rise times, and the remaining performance metrics were subsequently compared. In both methods, the same SMLTO with cutoff frequency 400 Hz and sigmoid-based switching function was used for load-torque estimation. Performance metrics were evaluated, and the final results are reported in Table 2.

**Table 2:** Selected metrics for simulation comparison of FCS-MPC and ES-DSPC method.

Method	Rise time [ms]	Speed RMSE [rpm]	Current THD [%]	$\text{ i}_{\text{ q } - \text{ ripple,rms }}$[%]
C-DSPC	55	11.3	14.48	9.97
ES-DSPC	55	14.2	14.02	12.12

The speed RMSE indicates that the C-DSPC achieves a lower tracking error (11.3 rpm) compared to the ES-DSPC (14.2 rpm). This suggests that, under the selected tuning, the ES-DSPC slightly compromises speed tracking accuracy in steady state. The increase in RMSE can be attributed to the additional decision constraints introduced by the sequential evaluation, which limit the available voltage vectors in each sampling interval. It should be emphasized that the speed RMSE can be further reduced by appropriate retuning of both controllers or by augmenting the cost function with an integral term.

The current harmonic distortion is marginally improved when using ES-DSPC. The total harmonic distortion of the stator current is reduced from 14.48 % for C-DSPC to 14.02 % for ES-DSPC. Although the improvement is modest, it indicates that the ES-DSPC can be competitive. A similar trend can be observed in the torque–producing current ripple. The value of the $i_{q-RMS}$ ripple increases from 9.97 % in C-DSPC to 12.12 % in ES-DSPC. This increase reflects a trade–off introduced by the enhanced sequential prediction strategy: while harmonic content is similar, the reduced flexibility in vector selection leads to a higher ripple in the torque–generating current component.

The primary objective is to investigate whether a comparable and competitive control performance can be achieved with a reduced number of weighting coefficients in the cost function, thereby simplifying the tuning process. The simulation results confirm that this objective is met. Despite the reduced tuning dimensionality, the ES-DSPC preserves identical dynamic performance in terms of rise time and achieves current harmonic distortion comparable to the conventional C-DSPC.

## Experimental results

**Fig. 3:**
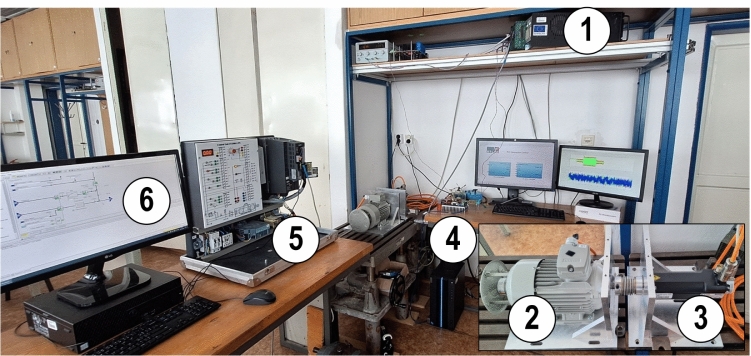
Experimental test bench, (1) OP 5600 rapid control prototyping controller, (2) load induction machine, (3) PMSM machine, (4) VSI INFINEON development kit, (5) loading industrial inverter, (6) PC for control of loading.

In this section, the performance of the proposed sequential DSPC method, along with its enhanced version, was analyzed under various operating conditions and compared against conventional DSPC. The experimental setup consisted of the OPAL-RT OP5600 used as rapid control prototyping controller, interfaced using RT-LAB 11.3.6 software. The control algorithms were developed and executed using MATLAB/Simulink R2017a. The experimental platform included a 1.16-kW PMSM by TG Drives, driven by the INFINEON EVAL-M1-IM818-A development board. The system operated at a sampling frequency of 40 kHz. An induction motor, driven through industrial frequency converter Siemens Sinamics G120, and parametrized via Siemens Starter V5.6 HF2 software was used as a load machine. The rotor position of the PMSM was measured using an incremental encoder with 1024 pulses per revolution. This configuration enables comprehensive validation of the control strategies under a variety of operating conditions. Key performance indicators, including steady-state accuracy, dynamic response, and total harmonic distortion (THD), were evaluated based on measured data. The experimental setup is shown in Fig. 3, and its parameters used for validation of all methods are listed in Tab. 3.

**Fig. 4:**
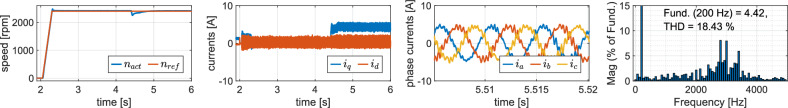
Experimental results, conventional DSPC with weighting factors $\lambda _\omega = 9$, $\lambda _{i_d} = 1$, $\lambda _{i_q} = 1$.

The conventional DSPC with empirically selected weighting factors was used as a performance reference for the proposed method. Fig. 4 shows the experimental results obtained using the conventional method, showing the measured speed, $q$-axis and $d$-axis currents, and spectral analysis. The speed reference is set to 80% of the nominal value, with a steep ramp-up and ramp-down of 250 ms. The dynamic response to a step load torque of 7 Nm can be observed in the speed and $q$-axis current, highlighting effective transient performance.

Relatively high current ripples and a total harmonic distortion (THD) of $18.43\%$ can be observed in Fig. 4. While the obtained THD value is relatively high, it is a consequence of the inherent properties of direct speed control structure evaluating speed and current within single objective function.

**Table 3:** Parameters of PMSM, type TGN3-0480-30-560 T1B, and predictive algorithm.

Symbol	Quantity	Value
$V_{DC}$	DC link voltage	560 *V*
$P_n$	nominal power	1162 *W*
$n_n$ [rpm]	nominal speed	3000 *rpm*
*p* [-]	pole pairs	5
$\psi _{pm}$	permanent magnet flux linkage	0.2267 *Wb*
*J*	motor inertia	0.00095 kgm$^2$
$R_{2ph}$	phase resistance	7.5 $\Omega$
$L_{2ph}$	phase inductance	22.7 *mH*
$T_s$	sampling time	25 $\mu$s
*m*	feedback gain of SMLTO	−80

### Experimental validation of S-DSPC

**Fig. 5:**
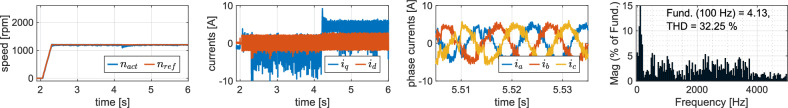
Experimental results, original sequential DSPC with sliding manifold constant $c = 0.1$.

The performance of S-DSPC was evaluated and compared under various operating conditions and with different values of the sliding manifold constant. The original S-DSPC implementation exhibited occasional instabilities and pronounced current ripple, as shown in Fig. 5, while their occurrence is largely independent of the selected value of the sliding manifold constant. In practice, it was not possible to operate this method with sliding manifold constant values above $c>0.5$, since higher values consistently led to unstable behavior. This limitation prevented further increase of *c* and restricted the achievable dynamic performance of the controller. Conversely, reducing *c* does not improve the performance of the controller enough to eliminate instabilities completely. Due to the instabilities of S-DSPC, it was not possible to achieve stable operation at higher speeds under the same load torque. Therefore, experiments were limited to 40% of the nominal speed.

Nevertheless, the experiments indicate that the sequential control principle itself is viable, as the controller is capable of regulating speed and responding to load disturbances, although with poor robustness and significant current ripple. This observation suggests that the main limitation lies in the original formulation rather than in the sequential concept itself, which motivated the development of the Enhanced S-DSPC approach, whose experimental results are presented in the following subsection.

### Experimental validation of Enhanced S-DSPC

**Fig. 6:**
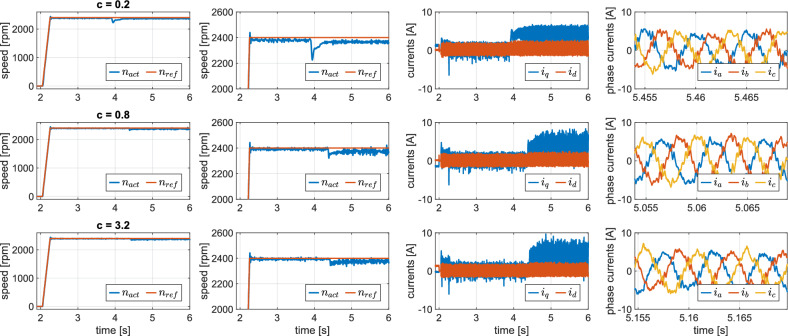
Experimental results, ES-DSPC ramp startup and loading for different sliding-manifold constants *c*.

Results of the enhanced version of sequential control can be observed in Fig. 6. To investigate the effect of the sliding-manifold term on both dynamic and steady-state performance, fast ramp changes in the reference speed and step load torque variations were applied for different values of the coefficient *c*. The values of *c* can now be raised, increasing the convergence of speed error and improve dynamic performance as intended. Instabilities were effectively eliminated, and as a result, current ripples were reduced compared to previous S-DSPC method. Notably, increasing *c* enhances the dynamic performance at the expense of increased current distortion. As expected, the improved dynamic performance comes at the cost of increased $q$-axis current peaks during loading and transient states.

**Fig. 7:**
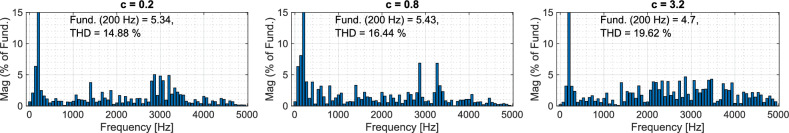
Spectral analysis for different values of *c* from Fig. 6.

Fig. 6 also illustrates improvement in the phase currents compared to the S-DSPC method. This is also confirmed in Fig. 7 showing spectral analysis of the phase currents under different values of constant *c*. Instabilities were eliminated and comparative values of THD with the conventional DSPC method were obtained. Table 4 summarizes the performance in experimental verification of the ES-DSPC method for different values of the sliding manifold constant *c*. For low values of the sliding manifold constant ($c = 0.2$), the controller exhibits the highest speed RMSE under both unloaded and loaded conditions, indicating limited corrective action of the sliding-mode-based term and reduced capability to reject load disturbances. In contrast, this setting results in the lowest current total harmonic distortion (THD) and the smallest ripple in the torque-producing current $i_q$, reflecting smoother current waveforms and less aggressive switching behavior.

**Table 4:** Selected metrics for experimental comparison of ES-DSPC method for different values of sliding manifold constant *c* for experimental results from Fig. 6.

Value	Speed RMSE - No load [rpm]	Speed RMSE - Loaded [rpm]	Current THD [%]	$\text{ i}_{\text{ q } - \text{ ripple,rms }}$[%]
c = 0.2	21.28	34.33	14.88	15.79
c = 0.8	10.64	23.71	16.44	20.04
c = 3.2	7.74	22.57	19.62	23.41

Increasing *c* to 0.8 significantly reduces speed RMSE under both operating conditions, demonstrating improved speed error suppression and enhanced robustness against load torque variations. This improvement is accompanied by a moderate increase in current THD and $i_q$ ripple, which can be attributed to more active control intervention.

For the highest tested value ($c = 3.2$), the controller achieves the lowest speed RMSE, particularly in the unloaded case, confirming that larger values of *c* accelerate speed convergence and strengthen disturbance rejection. However, this benefit comes at the expense of degraded current quality, as evidenced by the highest THD and current ripple.

The experimental results indicate that the sliding-manifold constant *c* fundamentally governs the compromise between speed-tracking accuracy and current quality in the ES-DSPC method. An intermediate range of *c* achieves an optimal balance, substantially reducing speed error while keeping current ripple and THD within acceptable limits. Decreasing *c* beyond this range does not lead to further meaningful THD reduction, limiting the practical benefits of this method.

**Fig. 8:**
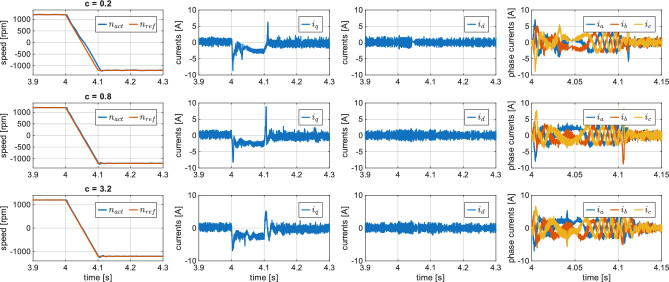
Experimental results, ES-DSPC speed reversal for different values of *c*.

To further evaluate the transient performance of the proposed ES-DSPC, additional experiments in Fig. 8 were conducted using a fast ramp speed reversal with a transition time of 100 ms. At the time instant, the rotor speed $n_{act}$ is reversed from 1200 rpm to $-1200$ rpm without load. It can be observed that increasing *c* leads to more accurate tracking of the speed reference during the transient, confirming the improved dynamic behavior of the controller.

## Conclusion

This paper investigated a sequential direct speed predictive control strategy for PMSM drives and analyzed its practical applicability. An enhanced formulation of the method was introduced to address stability and performance limitations observed in the baseline sequential approach. The study highlighted both the potential and the current limitations of sequential predictive control when applied to direct speed control. While the enhanced formulation improves operational robustness and enables simple controller tuning, a trade-off between the dynamic performance and the current ripple remains a key challenge. The findings indicate that, although sequential direct speed predictive control is a promising alternative to the conventional FCS-MPC approaches, further development is required to reduce distortion of the currents. This is especially important for higher-power systems, where requirements for current THD are very strict.

Future research will therefore focus on systematic THD reduction strategies and stability analysis. It is anticipated that further performance improvements could be achieved by extending the proposed control formulation with an additional degree of freedom; such as incorporating an integral term within the predictive control structure. A systematic investigation of this approach is left for future work. To the authors’ knowledge, this work represents an early experimental exploration of sequential direct speed predictive control for PMSM drives. Without significant improvements of sequential strategy, the benefits of proposed sequential direct speed control cannot outweigh performance of other predictive control strategies.

## Data Availability

The datasets generated during and/or analysed during the current study are available from the corresponding author on reasonable request.

## References

[1] Preindl, M. & Bolognani, S. Model predictive direct speed control with finite control set of PMSM drive systems. *IEEE Trans. Power Electron.* **28**, 1007–1015. 10.1109/TPEL.2012.2204277 (2013).

[2] Wang, F., Zhang, Z., Mei, X., Rodríguez, J. & Kennel, R. Advanced control strategies of induction machine: Field oriented control, direct torque control and model predictive control. *Energies* **11**, 10.3390/en11010120 (2018).

[3] Holtz, J. Advanced PWM and predictive control-An overview. *IEEE Trans. Ind. Electron.* **63**, 3837–3844. 10.1109/TIE.2015.2504347 (2016).

[4] Li, T. et al. Finite-control-set model predictive control of permanent magnet synchronous motor drive systems-An overview. *IEEE/CAA Journal of Automatica Sinica* **9**, 2087–2105. 10.1109/JAS.2022.105851 (2022).

[5] Vazquez, S., Rodriguez, J., Rivera, M., Franquelo, L. G. & Norambuena, M. Model predictive control for power converters and drives: Advances and trends. *IEEE Trans. Ind. Electron.* **64**, 935–947. 10.1109/TIE.2016.2625238 (2017).

[6] Cortes, P., Kazmierkowski, M. P., Kennel, R. M., Quevedo, D. E. & Rodriguez, J. Predictive control in power electronics and drives. *IEEE Trans. Ind. Electron.* **55**, 4312–4324. 10.1109/TIE.2008.2007480 (2008).

[7] Rodriguez, J. & Cortes, P. *Model Predictive Control*, vol. 1, chap. 3, 31–39 (John Wiley & Sons, 2012).

[8] Xu, Z., Wang, Z., Wang, X. & Cheng, M. Predictive current control method for dual three-phase PMSM drives with reduced switching frequency and low-computation burden. *IET Electric Power Applications* **14**, 668–677. 10.1049/iet-epa.2019.0529 (2020).

[9] M, D., Samithas, D., Kumar B, P. & Shitharth. Experimental analysis of enhanced finite set model predictive control and direct torque control in SRM drives for torque ripple reduction. *Scientific Reports* **14**, 10.1038/s41598-024-65202-1 (2024). 10.1038/s41598-024-65202-1 PMC11263375 39039123

[10] Fu, R. & Cao, Y. Hybrid flux predictor-based predictive flux control of permanent magnet synchronous motor drives. *IET Electric Power Applications* **16**, 472–482. 10.1049/elp2.12168 (2022).

[11] Tu, W., Luo, G., Chen, Z., Cui, L. & Kennel, R. Predictive cascaded speed and current control for PMSM drives with multi-timescale optimization. *IEEE Trans. Power Electron.* **34**, 11046–11061. 10.1109/TPEL.2019.2897746 (2019).

[12] Zhou, W., Song, Z., Xiao, X., Guo, Y. & Mo, Y. Sliding mode speed control for PMSM based on model predictive current. *Electronics* **13**, 10.3390/electronics13132561 (2024).

[13] Kumar, V., Sharma, V., Naresh, R. & Arya, Y. A novel predictive optimal control strategy for renewable penetrated interconnected power system. *Optimal Control Applications and Methods* **45**, 2190–2205. 10.1002/oca.3144 (2024).

[14] Zhang, Z., Liu, Y., Liang, X., Guo, H. & Zhuang, X. Robust Model Predictive Current Control of PMSM Based on Nonlinear Extended State Observer. *IEEE Journal of Emerging and Selected Topics in Power Electronics* **11**, 862–873. 10.1109/JESTPE.2022.3192064 (2023).

[15] Zhang, Z., Wang, X. & Xu, J. Robust Amplitude Control Set Model Predictive Control With Low-Cost Error for SPMSM Based on Nonlinear Extended State Observer. *IEEE Transactions on Power Electronics* **39**, 7016–7028. 10.1109/TPEL.2024.3380577 (2024).

[16] Zhang, Z., Guo, H. & Liu, Y. DC-Link Voltage Constraint Strategy for DC Power Supply Film-Capacitor Drive System Based on Improved Model Predictive Control. *IEEE Transactions on Industrial Electronics* **69**, 9849–9859. 10.1109/TIE.2022.3150105 (2022).

[17] Liu, M., Chan, K. W., Hu, J., Xu, W. & Rodriguez, J. Model predictive direct speed control with torque oscillation reduction for PMSM drives. *IEEE Trans. Ind. Informat.* **15**, 4944–4956. 10.1109/TII.2019.2898004 (2019).

[18] Gao, X., Abdelrahem, M., Hackl, C. M., Zhang, Z. & Kennel, R. Direct predictive speed control with a sliding manifold term for PMSM drives. *IEEE Trans. Emerg. Sel. Topics Power Electron.* **8**, 1258–1267, 10.1109/JESTPE.2019.2923285 (2020).

[19] Pancurák, L., Jure, T. & Kyslan, K. Finite control set model predictive direct speed control of PMSM. In *EDPE*, 2023 International Conference on Electrical Drives and Power Electronics, 396–401, 10.1109/EDPE58625.2023.10274055 (2023).

[20] Shahid, M. et al. Optimal weighting factor design based on entropy technique in finite control set model predictive torque control for electric drive applications. *Scientific Reports* **14**, 10.1038/s41598-024-63694-5 (2024). 10.1038/s41598-024-63694-5 PMC11150273 38834768

[21] Abu-Rub, H., Malinowski, M. & Al-Haddad, K. *Model Predictive Speed Control of Electrical Machines*, vol. 1, chap. 19, 608–629 (Wiley-IEEE Press, 2014).

[22] Anilkumar, M., Padhiyar, N. & Moudgalya, K. Lexicographic optimization based MPC: Simulation and experimental study. *Computers & Chemical Engineering* **88**, 135–144. 10.1016/j.compchemeng.2016.02.002 (2016).

[23] Shahid, M. B. et al. Hysteresis based predictive torque control without weighting factors for induction motor drives. *IET Control Theory & Applications* **18**, 2675–2692. 10.1049/cth2.12681 (2024).

[24] Norambuena, M. et al. A very simple strategy for high-quality performance of AC machines using model predictive control. *IEEE Trans. Power Electron.* **34**, 794–800. 10.1109/TPEL.2018.2812833 (2019).

[25] Zhang, Y., Zhang, B., Yang, H., Norambuena, M. & Rodriguez, J. Generalized sequential model predictive control of IM drives with field-weakening ability. *IEEE Trans. Power Electron.* **34**, 8944–8955. 10.1109/TPEL.2018.2886206 (2019).

[26] Zhang, K. et al. Tolerant sequential model predictive direct torque control of permanent magnet synchronous machine drives. *IEEE Trans. Transport. Electrific.* **6**, 1167–1176. 10.1109/TTE.2020.3008828 (2020).

[27] Davari, S. A., Norambuena, M., Nekoukar, V., Garcia, C. & Rodriguez, J. Even-handed sequential predictive torque and flux control. *IEEE Trans. Ind. Electron.* **67**, 7334–7342. 10.1109/TIE.2019.2945274 (2020).

[28] Sun, Z. et al. Weighting-factor-less model predictive control with multiobjectives for three-level hybrid ANPC inverter drives. *IEEE Trans. Emerg. Sel. Topics Power Electron.* **11**, 4726–4738, 10.1109/JESTPE.2023.3297191 (2023).

[29] Tang, Y., Xu, W., Dong, D., Liu, Y. & Ismail, M. M. Low-complexity multistep sequential model predictive current control for three-level inverter-fed linear induction machines. *IEEE Trans. Ind. Electron.* **70**, 5537–5548. 10.1109/TIE.2022.3192688 (2023).

[30] Liu, T., Yao, X., Wang, J. & Ma, C. Efficient two-vector-based sequential model predictive control for IM drives. *IEEE Trans. Emerg. Sel. Topics Power Electron.* **12**, 903–912, 10.1109/JESTPE.2023.3334468 (2024).

[31] Zerdali, E., Rivera, M. & Wheeler, P. A review on weighting factor design of finite control set model predictive control strategies for AC electric drives. *IEEE Trans. Power Electron.* **39**, 9967–9981. 10.1109/TPEL.2024.3370550 (2024).

[32] Zerdali, E., Kilic, C., Riccio, J., Rivera, M. & Wheeler, P. Sequential direct speed predictive control without weighting factors. In *PEMD 2024*, vol. 2024 of *13th International Conference on Power Electronics, Machines and Drives*, 167–171, 10.1049/icp.2024.2153 (2024).

[33] Karamanakos, P. & Geyer, T. Guidelines for the design of finite control set model predictive controllers. *IEEE Transactions on Power Electronics* **35**, 7434–7450. 10.1109/TPEL.2019.2954357 (2020).

[34] Pancurák, L. & Kyslan, K. Sensitivity analysis of sequential direct speed predictive control of PMSM. In *2025 International Conference on Electrical Drives and Power Electronics (EDPE)*, 293–298, 10.1109/EDPE66853.2025.11224112 (2025).

[35] Henwood, N., Malaizé, J. & Praly, L. A robust nonlinear Luenberger observer for the sensorless control of SM-PMSM: Rotor position and magnets flux estimation. In *IECON 2012 - 38th Annual Conference on IEEE Industrial Electronics Society*, 1625–1630 10.1109/IECON.2012.6388732 (2012).

[36] Li, T., Sun, X., Yao, M., Guo, D. & Sun, Y. Improved finite control set model predictive current control for permanent magnet synchronous motor with sliding mode observer. *IEEE Trans. Transport. Electrific.* **10**, 699–710. 10.1109/TTE.2023.3293510 (2024).

[37] Gong, C., Hu, Y., Ni, K., Liu, J. & Gao, J. SM load torque observer-based FCS-MPDSC with single prediction horizon for high dynamics of surface-mounted PMSM. *IEEE Trans. Power Electron.* **35**, 20–24. 10.1109/TPEL.2019.2929714 (2020).

[38] Liu, L., Song, H. & Liang, D. High performance sensorless control of PMSM with sliding mode load torque observer. In *2020 4th International Conference on HVDC (HVDC)*, 1060–1065, 10.1109/HVDC50696.2020.9292881 (2020).

[39] Cortes, P., Rodriguez, J., Silva, C. & Flores, A. Delay compensation in model predictive current control of a three-phase inverter. *IEEE Transactions on Industrial Electronics* **59**, 1323–1325. 10.1109/TIE.2011.2157284 (2012).

